# Recent Advances of Polyaniline-Based Biomaterials for Phototherapeutic Treatments of Tumors and Bacterial Infections

**DOI:** 10.3390/bioengineering7030094

**Published:** 2020-08-13

**Authors:** Chiranjeevi Korupalli, Poliraju Kalluru, Karthik Nuthalapati, Naresh Kuthala, Suresh Thangudu, Raviraj Vankayala

**Affiliations:** 1 Department of Chemical Engineering, National Tsing Hua University, Hsinchu 30013, Taiwan; chiranjeevikorupalli@gmail.com; 2 Department of Chemistry, University of Calgary, Calgary, AB T2N1N4, Canada; raju.poli@gmail.com; 3 Department of Chemistry, National Tsing Hua University, Hsinchu 30013, Taiwan; karthik.iitm904@gmail.com (K.N.); knareshiitm@gmail.com (N.K.); suresh120689@gmail.com (S.T.); 4 Department of Bioscience and Bioengineering, Indian Institute of Technology Jodhpur, Jodhpur, Rajasthan 342037, India

**Keywords:** polyaniline, photothermal therapy, cancer, bacteria

## Abstract

Conventional treatments fail to completely eradicate tumor or bacterial infections due to their inherent shortcomings. In recent years, photothermal therapy (PTT) has emerged as an attractive treatment modality that relies on the absorption of photothermal agents (PTAs) at a specific wavelength, thereby transforming the excitation light energy into heat. The advantages of PTT are its high efficacy, specificity, and minimal damage to normal tissues. To this end, various inorganic nanomaterials such as gold nanostructures, carbon nanostructures, and transition metal dichalcogenides have been extensively explored for PTT applications. Subsequently, the focus has shifted to the development of polymeric PTAs, owing to their unique properties such as biodegradability, biocompatibility, non-immunogenicity, and low toxicity when compared to inorganic PTAs. Among various organic PTAs, polyaniline (PANI) is one of the best-known and earliest-reported organic PTAs. Hence, in this review, we cover the recent advances and progress of PANI-based biomaterials for PTT application in tumors and bacterial infections. The future prospects in this exciting area are also addressed.

## 1. Introduction

Hyperthermia (also known as thermotherapy or thermal therapy) is a medical treatment approach that involves the exposure of the body tissue to higher temperatures in an effort to tackle various diseases such as cancer, bacterial infection, inflammatory diseases, etc., via eradication of disease cells or pathogens through the denaturation of proteins or disintegration of membranes [1,2,3,4]. The conventional hyperthermia methods involve induction heating, direct application of heat through the use of heated saline pumped through catheters, sitting in a hot room or wrapping a patient in hot blankets, resistive heating, microwave heating, ultrasound heating etc. [5,6]. However, most of these methods are invasive and may cause unwanted damage to healthy tissues [7]. Therefore, to overcome the limitations of conventional hyperthermia, noninvasive hyperemia methods involving the excitation of near-infrared (NIR) light, radiofrequencies, or inductively coupled magnetic fields have been employed to localize the generated heat to the diseased tissue [8].

Photothermal therapy (PTT) relies on the conversion of electromagnetic radiation energy into heat through the activation of photosensitizing agents and has drawn great attention in recent times owing to its minimal invasiveness, reduced side effects, and high specificity to diseased tissues [9]. In PTT, the NIR light with wavelength ranging from 700–900 nm is more useful when compared to that of the UV-Vis light, because biological chromophores (hemoglobin, oxyhemoglobin, and melanin) and water absorb strongly in this region, and convert to heat, thereby results in hyperthermia damage to both diseased sites and healthy tissues [10]. Additionally, NIR light penetrates deeper into biological tissues than UV-Vis light [10]. Therefore, it represents an ideal phototherapeutic agent (PTA) with large absorption cross-sections in the NIR region along with low toxicity, ease of functionalization, and high solubility in biocompatible solutions. In the last two decades, the advancements in the nanotechnology have led to the development of variety of inorganic PTAs with strong absorption in NIR region including nanostructures of noble metals such as Au, Ag or Pt, transition metal oxide or sulfide nanoparticles, carbon-based nanomaterials such as carbon nanotubes and graphene [11,12,13]. All these inorganic PTAs exhibited excellent photothermal therapeutic efficacies in in vitro and preclinical animal experiments; however, their poor biocompatibility, non-degradability, and long-term toxicity severely hinder their prospects in clinical applications.

Alternatively, organic nanoparticles such as conjugated polymer nanoparticles based on polyaniline (PANI), poly (3,4-ethylenedioxythio-phene): poly(4-styrenesulfonate), polypyrrole, polydopamine and semiconducting polymer nanoparticles, and porphysome have been developed as the promising candidates for PTT because of advantages including good biocompatibility, biodegradability, ease of surface modification and processing into nanoparticles with different size, and suitability for preparing multifunctional nanoparticles by co-loading with other diagnostic and therapeutic agents [14,15,16,17,18,19]. Conjugated polymers possess alternative single and double bonds in which unpaired electrons (π electrons) of carbon are localized in a p_z_ orbital that is positioned out of plane. These π electrons are mobile and liable to delocalization and transition. Upon excitation with photon energy, these π electrons undergo internal conversion (IC) to lowest singlet excited state (S1) and relax to the ground state via nonradiative pathways and can generate heat [20,21,22]. Among these organic PTAs, PANI was the first reported organic polymer PTA and has been widely applied in PTT and photoacoustic imaging (PAI) [14,23]. This review mainly discusses the recent advancements in the development of PANI-based biomaterials for photothermal ablation of tumors and pathogens.

## 2. Chemical Structure and Stability of PANI

**Figure 1:**
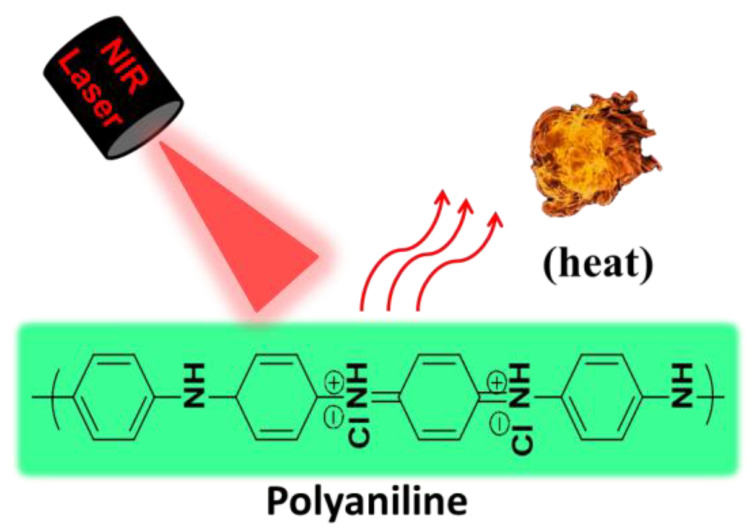
Schematic illustration for photothermal property of PANI.

PANI, with a ratio of diiminoquionoid and diaminobenzenoid rings, is a well-known conducting polymer and has attracted great attention in biomedical applications due to its low cost, environmental and chemical stability, facile synthesis, high conductivity, and outstanding physicochemical properties [24,25,26,27]. Furthermore, PANI exhibits the unique feature of switching between a conductor and an insulator depending on the extent of oxidation (variation in the number of electrons) and the degree of protonation (variation in the number of protons). Among different oxidation states, emeraldine base (EB) and emeraldine salt (ES) are the most stable oxidation states of PANI, and they are inter-convertible to each other through protonation and deprotonation based on the pH of the microenvironment [28]. Switching from the EB state to the ES state in the presence of oxidative species or an acidic environment leads to a red shift of PANI’s absorption peak from the visible to the NIR region due to charge transmission between benzenoid and quinoid rings through enhanced electron movement, leading to potential PTT applications [14,29]. Therefore, PANI has been exploited in PTT applications owing to its biocompatibility and high photothermal conversion efficiency [14,29,30,31]. Unlike organic dyes, PANI exhibits excellent photostability upon light irradiation, thus enabling repeated phototherapeutic treatments [28]. Nevertheless, the hydrophobic nature and the necessity of extremely low pH (pH < 4) conditions for EB to ES transition significantly hinders the biological applications of PANI [32]. Thus, researchers have made tremendous efforts to enhance the solubility of PANI in biological media in order to make PANI useful as PTA in the pathological environment for the ablation of cancer cells or bacteria (Figure 1).

## 3. PANI-Based Biomaterials for Tumor Ablation

Cancer is a major cause of death and accounts for one in every four deaths in the United States [33]. It is a disease that involves uncontrolled growth of abnormal cells and has the potential to spread or invade to organs of the body [34]. Conventional therapeutic treatments for cancer include surgical excision, radiotherapy, chemotherapy, and combination methods [35,36,37]. However, surgery usually results incomplete tumor removal, whereas radiotherapy and chemotherapy cause systemic cytotoxicity due to non-specific drug delivery to all tissues, including healthy tissues [38]. The above-mentioned drawbacks necessitate the development of new therapeutic approaches to efficiently eliminate cancer cells without damaging the healthy tissues. It is known that cancer cells are vulnerable to temperatures above 45 °C. Thus, PTT, as a non-invasive hyperthermia approach, is gaining greater attention for the treatment of tumors, because it has the capability to selectively destruct cancerous cells without damaging surrounding healthy tissue [10]. Therefore, in this section we discuss developments in PTT of tumors using PANI-based materials. The reported PANI-based PTAs for cancer treatment are summarized in Table 1.

### 3.1. Synthesis of PANI PTAs

#### 3.1.1. Chemical Polymerization

**Figure 2:**
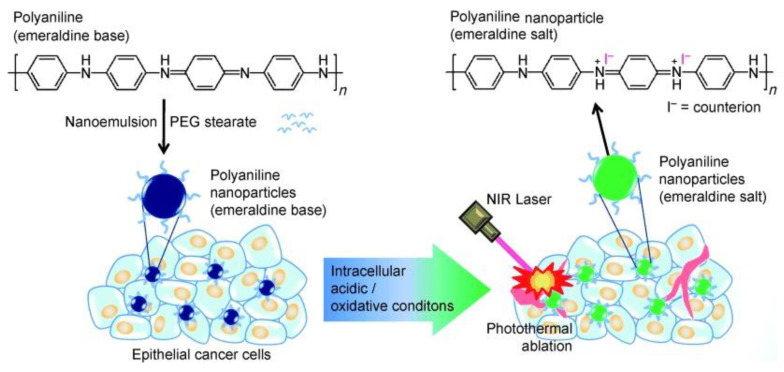
Schematic illustration of the preparation of polyaniline nanoparticles and their application for the photothermal ablation of epithelial cancer cells by NIR laser irradiation (image is reproduced from [14] with the copyright permission from WILEY-VCH Verlag GmbH & Co.).

The photothermal conversion capability of PANI was first published by Yang et al. in 2011 [14]. This is also the first demonstration on the usage of organic nanoparticles for PTT applications. In this study, hydrophobic PANI was synthesized via chemical oxidative polymerization using protonated aniline monomer and ammonium persulfate (APS) as an oxidant followed by dedoping with alkali solution. Then, the hydrophobic EB polymer was coated with PEGylated fatty acid through the nanoemulsion method to provide required water solubility. The as-developed PANI NPs demonstrated colloidal stability in biological media, and intracellular pH and oxidative environment dependent NIR absorption. Furthermore, PANI NPs transformed into EB state to NIR light absorbed ES state by biological doping process and caused hyperthermic ablation of A431 tumor cells in both in vitro and in vivo experiments via PTT effect (Figure 2). However, these nanoparticles are larger in size, with a mean diameter of 115.6 nm, and the toxicity under dark conditions was also not well studied. Thus, several other research groups have synthesized PANI NPs using various biocompatible polymers as surfactants and synthetic strategies to improve PANI’s dispersibility in biological media and decrease the size of NPs [14,30,39,40,41,42]. In one study, Zhou et al. synthesized PANI NPs with suitable size through hydrothermal method using oxidant ammonium persulfate (APS), and stabilizers 6-aminocaproic acid sodium oleate. The as-synthesized PANI NPs were surface-modified with polyoxyethylene chains containing pluronic F-127 for hydrophilic conversion (F-PANPs). The F-PANPs exhibited good water-solubility, size with a mean diameter of 48.5 nm, high photothermal conversion efficiency and therapeutic effect [30]. In another study, PANI nanoparticles were prepared through nucleation and growth polymerization, using poly (vinylpyrrolidone) (PVP) as a surfactant and APS as oxidant [39,40]. The as synthesized PANI NPs did not induce significant toxicity to the cells under dark conditions indicating the biocompatibility of PANI. This study also suggests that cell toxicity after laser irradiation could be attributed to a synergistic effect mediated by hyperthermia-induced cell necrosis and heat diffusion/ROS migration caused apoptosis [39].

Recently, Wang et al. developed a porous metal organic framework hybrid (MOF) as a PTA by coating PANI onto UiO-66 (UiO-66@PAN) through chemical oxidation [41]. The as-synthesized UiO-66@PAN showed good strong NIR absorbance and photothermal properties. Additionally, the UiO-66@PAN effectively caused cancer cell death and tumor growth inhibition in in vitro and in vivo, respectively, upon laser irradiation. By using this approach, one can combine drug delivery with PTT which is difficult to achieve with previously discussed studies.

#### 3.1.2. Enzyme-Catalyzed Polymerization

Usually, the polymerization of PANI involves using chemical and electrochemical methods which are either environmentally hazardous or produces water-insoluble products that require further modification with surfactants [14,30]. These concerns can be overcome by using enzyme-catalyzed polymerization approach [62,63]. For example, Li et al. reported an environmentally friendly approach to produce water soluble PANI nanoparticles using eco-friendly oxidant hydrogen peroxide, peroxidase activity mimic iron phosphates (FePOs) and polystyrene sulfonate (PSS) as template [42]. The as-synthesized PANI nanoparticles demonstrated good water solubility and remarkable tumor cells killing effect on HeLa via the PTT mechanism. This approach has paved a way for the synthesis of PANI in eco-friendly manner.

### 3.2. Multifucntional PANI-Based Materials

The studies discussed above successfully overcame several hurdles of PANI for applications in PTT, e.g., water solubility and NIR photothermal conversion efficiency. However, these PANI NPs considerably exhibit a single function, i.e., PTT, and lack other functionalities intended to improve delivery efficiencies, therapeutic efficacy, and, ultimately, patient outcome. It is known that multifunctional nanomedicine platforms with targeting, imaging and therapeutic functionalities can improve the overall treatment efficacies and can eventually help to minimize the damage of normal human tissues during treatment [64,65,66]. Therefore, researchers have made extensive efforts to develop multifunctional PANI-based materials by employing various bioactive molecules, polymers, etc., to empower PANI-based materials with targeting, imaging properties along with PTT.

#### 3.2.1. PANI Nanoparticles with MRI Imaging and PTT

**Figure 3:**
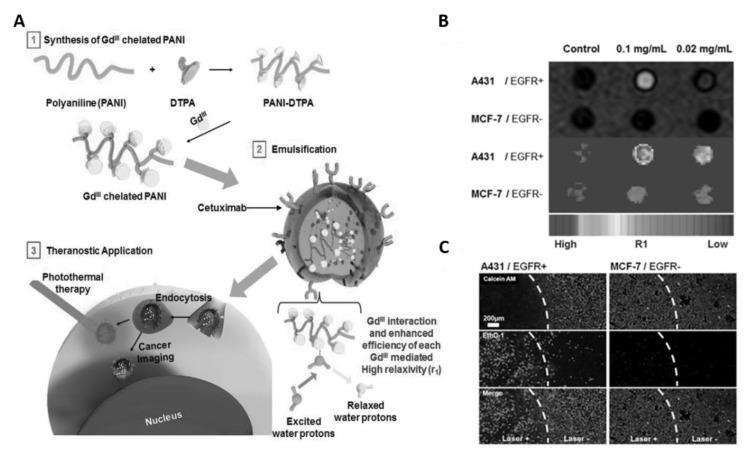
(**A**) Schematic illustration for the preparation of Gd (III)-loaded polyaniline nanoparticles (GPAPs) and their application as theranostic agent for epithelial cancer. (**B**) T_1_-mapped and respective color-mapped MR images of A431 (EGFR+) and MCF-7 (EGFR−) cell lines after treatment with different amount of GPAPs. (**C**) Fluorescence microscopic images of A431 (EGFR+) and MCF-7 (EGFR−) cells stained with Calcein AM and ethidium homodimer-1 (EthD-1) after treatment with GPAPs followed by NIR laser irradiation for 10 min (808 nm, 10 W cm^−2^). White-dotted curves represent the location of the laser beam (images are reproduced from [43] with the copyright permission from WILEY-VCH Verlag GmbH & Co.).

Magnetic resonance imaging (MRI) is a non-invasive imaging technology which allows the examination of anatomic structures, physiological functions, and molecular composition of tissues. It is often used for prognosis, and to monitor the activity and optimal treatment response of disease [67,68]. Additionally, it is the most reliable imaging technique for the diagnosis of tumors due to its high spatial resolution, superior soft tissue contrast and specificity, and good penetration depth [69]. Therefore, by combining MRI with therapeutic modalities, one can achieve tumor imaging and treatment at the same time. To this end, in an early study, Lee et al. developed cetuximab (CET; anti EGFR) modified and gadolinium (Gd), an effective MR contrast agent, enriching PANI NPs (GPAPs) as theranostic agents to achieve tumor targeting, MRI and PTT simultaneously through a single nanosystem [43]. In this nanosystem CET, Gd and PANI endow the GPAPs with epithelial cancer cell targeting, MRI imaging, and PTT properties, respectively (Figure 3A). The results from in vitro experiments clearly demonstrated that high epidermal growth factor receptor (EGFR) expressed A431 cells have efficiently uptaken GPAPs due to CET and EGFR interactions and exhibited strong and bright signals in the T_1_-weighted MR-images than untreated A431 cells; whereas, low EGFR expressed MCF-7 cells have minimal uptake of GPAPs due to lack of specific interactions, and thus did not exhibit any noticeable differences when compared to untreated MCF-7 cells in terms of T_1_-weighted MR-images signal intensities (Figure 3B). Furthermore, upon NIR-irradiation, significant cell death was observed only for GPAPs treated A-431 cells compared to untreated A-431, MCF-7 and GPAPs treated MCF-7 cells (Figure 3C). These results reveal that GPAPs are useful to target EGFR express cells, MRI imaging and PTT. This study had exposed a pathway to develop PANI-based multifunctional materials with imaging and PTT modalities. For instance, Lin et al. developed Cu-doped PANI (CuPANI) nanoshuttles (NSs) as theranostic agents by doping Cu (II) ions into PANI NSs [44]. The Cu ions and PANI allowed the CuPANI NSs with MRI imaging and PTT properties, respectively.

Usually, PANI molecules exhibit strong affinity between themselves due to aromatic π-π interactions and interchain hydrogen bonding, thus hindering their hybridization with inorganic materials [69,70]. To overcome this limitation, Lee et al. used pyrene as a crosslinker between PANI and MnFe_2_O_4_ magnetic nanoparticles and synthesized magnetic polyaniline nanoparticles (MPANs) for simultaneous MRI imaging and PTT. The MPANs were conjugated with CET which further allowed targeting ability [45]. This study demonstrated a pathway for the fabrication of composites between PANI and inorganic substances thereby achieve multi-functionality to the nanoparticles.

#### 3.2.2. PANI Nanoparticles with PAI Imaging and PTT

Photoacoustic imaging (PAI) is a noninvasive and nonionizing biomedical imaging modality and based on the use of laser-generated ultrasound [71]. It possesses high optical contrast and spatial resolution, and is promising for diagnosis of disease. PAI has high sensitivity to optical absorption and deep tissue penetration than other optical imaging technologies such as fluorescence imaging [72]. Thus, combining PAI with PTT can allow a precise diagnosis and excellent therapeutic efficacy of tumor. Wang et al. developed lipid-PANI hybrid nanoparticles for PA imaging guided PTT of tumor. Folic acid (FA), a targeting ligand, was also conjugated to lipid-PANI nanoparticles (FA-Lipid-PANI NP) to achieve tumor targeting property [46]. The FA-Lipid-PANI NPs demonstrated significant PAI signals and PTT effect in in vivo upon laser irradiation due to high NIR absorbance. Later on, ICG-Ag@PANI [47], PANI/Si/HA-DA [45], PANI/γ-PGA [23] nanocomposites were reported for PAI imaging mediated PTT. All these composites exhibited precise diagnosis and PTT effect of tumor upon laser irradiation. In a continuation of above studies, Jiang et al. synthesized hyaluronic acid (HA)-PANI NPs through electrostatic interactions between negatively charged HA and positively charged PANI [49]. The as-synthesized HA-PANI NPs exhibited targeting specificity to CD-44 expressed cancer cells and PTT mediated cell-killing efficacy both in vitro and in vivo.

### 3.3. Self-Doping of PANI

**Figure 4:**
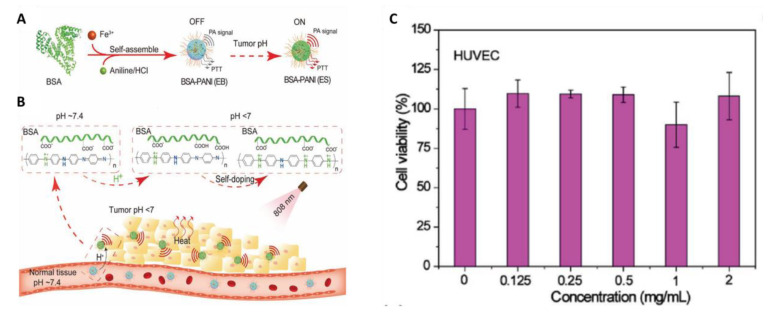
(**A**) Schematic illustration for the preparation bovine serum albumin (BSA)-polyaniline (PANI) assemblies. (**B**) BSA-PANI assemblies for amplified photoacoustic imaging and augmented photothermal therapy. The potential mechanism is based on intermolecular acid–base reactions between carboxyl groups of BSA and imine moieties of PANI. (**C**) In vitro biocompatibility of the BSA-PANI assemblies for noncancerous HUVECs cells after 24 h of incubation. (images are reproduced from [31] with the copyright permission from WILEY-VCH Verlag GmbH & Co.).

It is well known that PANI exists in a dedoped EB state in the physiological pH environment and converts from the EB state to the ES state under acidic conditions through protonation, which causes a redshift in absorption from visible to NIR region and improves its applicability for PTT applications [29]. This conversion requires an acidic environment with pH < 4.0; therefore, the tumor acidic microenvironment (pH 5.4–7.0) is insufficient for this conversion and severely limits the application of PANI as PTA for cancer treatment [32]. It was reported that pH response range of PANI can be tuned by self-doping of PANI through introduction of acidic groups into the PANI chains [73] or by improving the charge transfer rate via incorporation of conductive nanomaterials into PANI matrix [74]. When relying on this strategy, researchers have made attempts to improve the application of PANI as PTA in cancer treatment [31,50,51,52]. Hong and coworkers synthesized PANI nanoparticles by incorporating lauric acid (LA) as a stabilizer and localized dopant [50]. These nanoparticles exhibited high photothermal conversion efficiency even at a neutral pH due to doping with LA. In another study, 3-mercapto-1-propanesulfonic acid was grafted onto the PANI backbone to avail with self-doping property [51]. All the above mentioned attempts to tune the pH response range of PANI are based on the introduction of acid groups to the PANI backbone. However, the acid self-doped PANI was mostly soluble in alkaline conditions only, which is limiting its suitability for biological applications. Later on, Qu et al. introduced gold nanoparticles (AuNPs) into the core of PANI nanoparticles through the electrostatic interaction. These nanocomposites demonstrated pH-dependent NIR absorbance and preserved PTT effect in tumor microenvironment due to charge transfer between AuNPs and PANI [52]. Nevertheless, the introduction of nonbiodegradable Au may produce long-term toxicity. To minimize these limitations, Tian and coworkers recently developed a tumor pH environment-responsive PANI-based theranostic agent using bovine serum albumin (BSA) and PANI for PAI and PTT of tumors [31]. In this study, the intermolecular acid–base reactions between the imine moieties of the PANI backbone and the carboxyl groups of BSA led to a self-doping effect and change in the pH responsive range of PANI thereby redshift of absorption peak from visible to NIR region at a relatively high pH (<7.0) (Figure 4). Furthermore, the as-developed BSA-PANI NPs demonstrated strong PAI performance and PTT in tumor environment both in in vitro and in vivo [31]. The biocompatibility of BSA-PANI NPs was examined in vitro using non-cancer HUVEC cells. As shown in Figure 4C, the BSA-PANI NPs did not exhibit significant toxicity to HUVEC cells in dark even at high concentrations up to 2 mg/mL indicating that PANI-based PTAs are biocompatible [31]. These results can provide a possible pathway for the development of PANI-based materials that are responsive to pH of tumor.

### 3.4. Combination Therapy of Cancer Using PANI-Based Materials

In general, it is difficult to eradicate tumors completely using a single therapeutic modality. Thus, integration of different treatments with diverse strategies, such as chemotherapy, radiotherapy (RT), photodynamic therapy (PDT) and PTT into single platform has attracting great interest in recent times because it can enhance the therapeutic outcome of cancer [75]. Various bioactive molecules can be incorporated into PANI NPs through chemical interactions such as grafting onto functional groups or physical interactions including electrostatic, π-π stacking, hydrogen bonding or hydrophobic interactions. Therefore, it is a suitable PTA to combine with other modalities. In this regard, PANI has been increasingly integrating with other therapeutic biomolecules to achieve high curative outcome through synergetic effect between PTT and other therapeutic modalities.

#### 3.4.1. PANI Nanocomposites with Chemotherapy and PTT

Chemotherapy is a conventional approach in the treatment of cancer. However, undesired side effects, discomfort of patients, and multi-drug resistance of cancers limit its curative efficacy [76]. It has been reported that hyperthermia can sensitize the cells to chemotherapeutic drugs by affecting the DNA damaging process [77]. Therefore, a chemotherapeutic agent has been incorporating into NIR responsive materials to induce synergistic anticancer effects [78,79]. In a study, Nguyen and coworkers developed a multifunctional hybrid polymer system comprising of PANI NPs as PTA and Methotrexate (MTX) as an anticancer drug for combined photo–chemotherapy [53]. In in vivo experiments, mice treated with combined therapy showed a higher tumor growth inhibition rate than PTT or chemotherapy alone, due to a synergistic effect, implying that combining PANI with other chemotherapeutic agents can exert better efficacy. The other PANI-based chemo-photothermal agents are also listed in Table 1 [54,55,56,57,58].

#### 3.4.2. PANI Nanocomposites with RT and PTT

**Figure 5:**
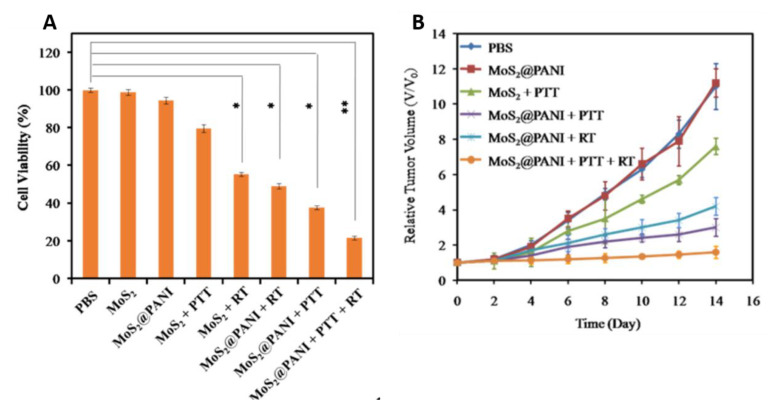
(**A**) Cell viabilities of 4T1 cells treated with PBS, MoS_2_, or MoS_2_@PANI with or without laser irradiation (808 nm, 1.5 W cm^−2^) and X-ray radiation (6 Gy). (**B**) Tumor growth in different groups of mice after various treatments (images are reproduced from [59] with the copyright permission from American Chemical Society).

Radiotherapy (RT), another cancer treatment approach along with surgery and chemotherapy, kills cancer cells and shrinks tumors under high doses of radiation, and is a cost-effective single modality treatment [35]. It involves local application of ionizing radiation (e.g., γ-ray, X-ray) on the tumor to generate oxygen free radicals from surrounding water molecules that cause the DNA damage [80]. The cellular oxygen levels highly influence the degree of cellular damage in this approach. Therefore, the tumor hypoxia is a major problem for implementation of RT [81]. However, a proper level of hyperthermia could improve oxygen levels in the tumor microenvironment by increasing intratumoral bloodstream thereby sensitize cells to RT [82,83]. Therefore, it has been reported that combining PTT with RT can improve the therapeutic outcome of tumors [84,85]. Thus, hybrid nanocomposites were developed by combining radio therapeutic agents and PANI for radiophotothermal therapy [59,60]. Wang et al. fabricated an inorganic–organic nanohybrid using MoS_2_ quantum dot and polyaniline (MoS_2_@PANI) to accomplish simultaneous CT/PA imaging and synergistic PTT/RT combination therapy for tumor [59]. In vitro, the as-fabricated MoS_2_@PANI nanohybrids induced 78.4% cancer cell death upon simultaneous PTT/RT treatments, whereas single PTT or RT induced 62.3% or 51.1% cell death, respectively (Figure 5A). In vivo, MoS_2_@PANI + PTT/RT group remarkably inhibited tumor growth compared to control groups, including PBS, MoS_2_ + PTT, MoS_2_@PANI and MOS_2_@PANI + PTT alone/RT alone (Figure 5B). These results demonstrate the efficiency of PANI in eradicating tumors through synergistic radio-photothermal therapy.

#### 3.4.3. PANI Nanocomposites with PDT and PTT

Photodynamic therapy (PDT) is a clinically approved non-invasive therapy that can be used in the selective treatment of various types of cancers [86]. It causes cell death through generation of reactive oxygen species (ROS) such as hydroxyl radical (OH), singlet oxygen (^1^O_2_), and superoxide (O2^−^) intracellularly, upon photoexcitation of photosensitizers with light of suitable wavelength [64]. However, the low oxygen levels in the tumor microenvironment are also the main obstacle in PDT [87]. Therefore, PDT has been combined with PTT to enhance the oxygen levels and improve therapeutic efficacy [88]. Most organic photosensitizers are hydrophobic and contain an aromatic component; therefore, they can be easily combined with PANI through hydrophobic or π-π stacking interactions to exert PDT and PTT modalities in a single nanoplatform [47,58,60]. For instance, Tan et al. loaded the photosensitizer Indocyanine Green (ICG) (the only FDA approved NIR dye) into Ag@PANI nanocomposites through π-π stacking and the hydrophobic interaction [47]. The as-synthesized ICG-Ag@PANI nanocomposite showed strong optical absorption in the NIR region and excellent photothrermal and ROS generation properties upon laser irradiation. In vitro and in vivo, the combination of PTT and PDT exhibited remarkable cancer cell lethality and tumor growth inhibition, respectively, compared to PTT or PDT alone upon photoexcitation of ICG-Ag@PANI with 808 nm light. This study is showed a way to incorporate photosensitizers with PANI.

Apart from this, PANI was also combined with immunotherapeutic drugs [61] through hydrophobic interactions to achieve PTT mediated immunotherapy. Overall, the results from these studies reveal that the therapeutic outcome can be enhanced using combinational therapy and necessitating further development of such strategies using PANI.

## 4. PANI-Based Biomaterials for Eradication of Bacterial Infections

Infectious diseases caused by pathogenic bacterial infections have become a major healthcare problems in human health and causing nearly one third of deaths globally [89,90]. The most common treatment for bacterial infections is usage of antibiotics. However, the overuse of antibiotics has resulted in the emergence of multidrug-resistant bacteria such as vancomycin-resistant enterococcus (VRE), vancomycin-resistant staphylococcus aureus (VRSA), and methicillin-resistant staphylococcus aureus (MRSA), which pose a serious threat to human health [91]. Therefore, alternative bactericidal therapeutic strategies for which microbes are less prone to develop resistance are urgently needed. Heat at temperatures above >50 °C can cause a bactericidal effect through denaturation of proteins/enzymes or cell membrane damage [92,93]. Thus, PTT has been considered one of the most promising novel bactericidal therapeutic approaches to combat MDR bacteria because it is less potential to stimulate resistance and toxicity. Thus, this section discusses the recent research findings of PANI-based materials for PTT eradication of bacterial infection and summarized in Table 2.

### 4.1. Self-Doping of PANI

**Figure 6:**
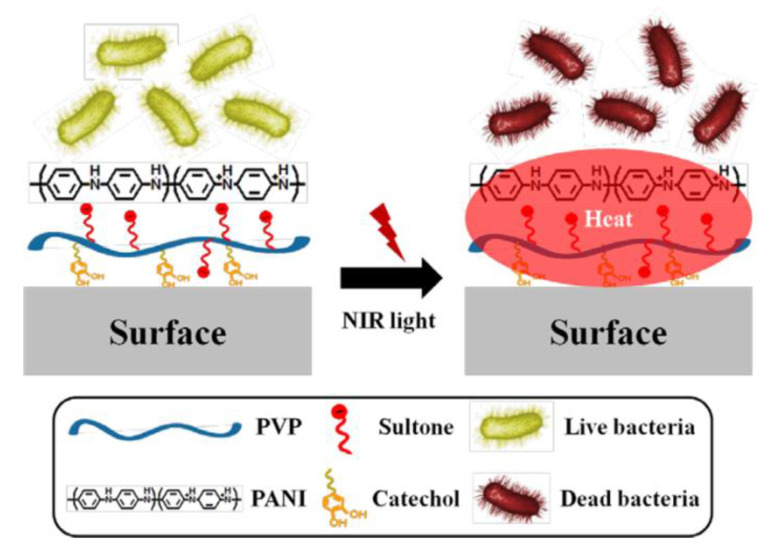
Schematic illustration for the preparation and application of PVPS:PANI coating and NIR irradiation for the photothermolysis of bacteria (images are reproduced from [94] with the copyright permission from American Chemical Society).

As we discussed before, PANI can exhibit strong photothermal conversion efficiency at pH below 3. Since the local pH of bacterial infection is likely to be between 6.0–6.5 [98], it is impossible for PANI to exhibit high light-to-heat conversion efficiency in an infection environment. Therefore, it is necessary to further modify PANI with self-doping agents such as acids to make PANI practical for bacterial eradication. Kim et al. developed a PANI-based photothermal agent using poly (vinylpyrrolidone) sulfobetaine (PVPS) and PANI (PVPS-PANI) for bacterial eradication [94]. The PVPS-PANI was formed through electrostatic interaction between negatively charged sulfones in PVPS and cationic PANI. These interactions also led to the charge transfer and consequently resulted in the improvement of the optical properties of PANI. Upon NIR laser exposure, PVPS-PANI exhibited increase in the solution temperature due to broad NIR absorption and eradicated both gram-positive and gram-negative bacteria through bactericidal effect (Figure 6).

### 4.2. Bacteria-Trageting and Multifucntinal PANI-Based Materials

**Figure 7:**
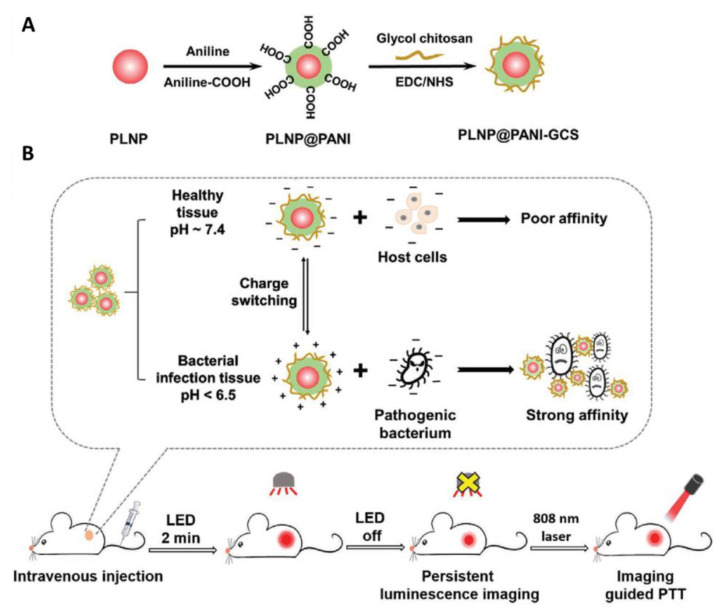
(**A**) Preparation of PLNP@PANI-GCS. (**B**) Illustration of PLNP@PANI-GCS for persistent luminescence imaging-guided photothermal therapy of bacterial infection (images are reproduced from [97] with the copyright permission from WILEY-VCH Verlag GmbH & Co.).

The uncontrollable distribution of PTT agents leads to a rise in the temperature of surrounding healthy tissue upon irradiation and causes damage to the normal cells. Therefore, to confine the heat generation to the infection site and selectively eradicate bacteria, pH-responsive physical state transformable and charge switchable nanoparticles have been developed using chitosan derivatives as backbone polymers and PANI as PTT agent [29,95]. Likewise, recently, Yan et al. fabricated a pH switchable nanoplatform composed of NIR-emitting persistent luminescence nanoparticles (PLNP; Zn_1.2_Ga_1.6_Ge_0.2_O_4_:Cr^3+^), PANI and glycol chitosan (GCS) for persistent luminescence imaging-guided selective eradication of pathological cells while protecting surrounding normal cells (Figure 7) [97]. The GCS and PANI endowed the PLNP@PANI-GCS nanoparticles with pH-responsive charge switchable property and PTT property, respectively. The as developed NPs caused aggregation of the bacteria at the pH of focal infection through electrostatic interactions and destroyed them without damaging surrounding normal cells upon laser irradiation due to strong PTT efficiency of PANI.

## 5. Summary and Future Perspectives

Photothermal therapy (PTT) has been proposed as a prospective alternative treatment for conventional therapeutic modalities of tumor and bacterial infections. Polyaniline (PANI) is a well-known and interesting organic photothermal agent (PTAs) owing to its unique properties. However, the inherent properties of PANI, which include hydrophobicity and low transition pH, severely limit its practical utility in PTT applications. This review summarized the recent advancements in the research on PANI that have been made to improve its applicability. The PANI surface has been modified with biocompatible polymers through nanoemulsion and chemical grafting methods to improve its dispersibility in biological media and its transition pH through self-doping. To further improve the specificity and therapeutic outcome, the photothermal conversion efficiency of PANI was combined with targeting moieties, imaging agents and other therapeutic modalities. Despite great achievements, there is still a lot of room to carry out research on PANI. The possible directions for future research are as follows: (1) detailed information on the long-term toxicity, bio-distribution, and biodegradation of PANI is missing. Such investigations are needed for potential clinical translation; (2) although the photothermal properties of PANI have been known for almost one decade, the number of reports based on the PTT properties of PANI is low; (3) one of the unique properties of PANI is its pH-responsive photothermal properties, which should be explored in more detail in the design of PTT, including therapeutic materials; and (4) most studies on PANI as PTA have been related to tumor treatment, and very few have been related to bacterial infection. It should also be considered for other applications such as for inflammatory diseases. We strongly believe that this review will shed light on the advancements and promising and exciting possibilities in the field of research on PTT using PANI-based materials, and we expect that this will be the subject of future research.

## Figures and Tables

**Table 1:** Summary of PANI-based photothermal agents for cancer treatments.

Composition of PTA	PTA Structure	Irradiation Conditions	In Vitro	In Vivo	Function	Reference
PANI/PEG	NPs	808, 2.45 W/cm^2^	A431	Yes	PTT	[14]
PANI/F-127	NPs	808, 0.5 W/cm^2^	HCT116	Yes	PTT	[30]
PANI/PVP	NPs	785, 0.5 W/cm^2^	LM2	No	PTT	[39,40]
UiO66@PANI	NPs	808, 0.7 W/cm^2^	CT26	Yes	PTT	[41]
PANI/PSS	NPs	808, 2.08 W/cm^2^	HeLa	No	PTT	[42]
PANI/Gd^III^/PVA/CET	NPs	808, 1.5 W/cm^2^	A431	Yes	Targeting, MRI, PTT	[43]
PANI/Cu(II)	NPs	808, 0.3 W/cm^2^	HeLa	Yes	MRI, PTT	[44]
PANI/MnFe_2_O_4_/ pyrene/CET	NPs	808, 5 W/cm^2^	A431	No	Targeting MRI, PTT	[45]
PANI/Lipid/FA	NPs	808, 2.0 W/cm^2^	Hela	Yes	PAI, PTT	[46]
PANI/Ag/ICG/ PEG	NPs	808, 1.0 W/cm^2^	Hela	Yes	PAI, FI, PTT, PDT	[47]
PANI/Si/HA-DA	NPs	808, 2.0 W/cm^2^	MDAMB-231 KB MDCK	Yes	FI, PTT	[48]
PANI/γ-PGA	NPs	808, 1.5 W/cm^2^	4T1	Yes	PAI, PTT	[23]
PANI/HA	NPs	808, 0.64 W/cm^2^	HFF HCT-116 HeLa	Yes	Targeting, PTT	[49]
PANI/LA/Tween80	NPs	808, 10 W/cm^2^	MDA-MB-231	No	Self-doping, PTT	[50]
NMPA/CS	Hydrogel	808, 0.5 W/cm^2^	Hep3B	Yes	Self-doping, PTT	[51]
PANI/Au/PEG	NPs	808, 2 W/cm^2^	HEK293T HepG2 HeLa	Yes	Charge transfer, PTT	[52]
PANI/BSA	NPs	808, 1.0 W/cm^2^	4T1	Yes	Self-doping, PAI, PTT	[31]
PANI/LT/MTX/PVP/SDS/PLGA/DSPE-PEG2000-mal	NPs	808, 2 W/cm^2^	MCF-7 MDA-MB-231	Yes	Targeting, Chemotherapy PTT	[53]
PANI/5-FU/ZIF-8	NPs	980, 0.8 W/cm^2^	MCF7	Yes	Chemotherapy, PTT	[54]
PANI/cisplatin/lecithin/cRGD or FA-PEG-DSPE	NPs	808, 1.54 W/cm^2^	MGC-803 MDA-MB-231	No	Targeting, Chemotherapy, PTT	[55]
PANI/DOX/Si	NPs	808, 1.4 W/cm^2^	4T1	Yes	Chemotherapy, PTT	[56]
PANI/cisplatin/lecithin/PEG-PCL/ Tmab	NPs	808, 1.54 W/cm^2^	SK-BR-3	No	Targeting, Chemotherapy, PTT	[57]
PANI/Ce6/lecithin/cRGD-PEG-PCL	NPs	808, 1.54 W/cm^2^	MGC-803 MCF-7	No	Targeting, Chemotherapy, PTT, PDT	[58]
PANI/MoS_2_/PEG	NPs	808, 1.5 W/cm^2^	4T1	Yes	PAI, CTI RT, PTT	[59]
PANI/WS2/Ce6/HA	NPs	808, 1.5 W/cm^2^	4T1	Yes	FI, PAI, CTI, PTT, PDT, RT	[60]
PANI/R848/GCS	NPs	808, 0.9 W/cm^2^	CT26	Yes	Immunotherapy, PTT	[61]

**Table 2:** Summary of PANI-based photothermal agents for bacterial infection treatment.

Composition of PTA	PTA Structure	Irradiation Conditions	Bacteria	In Vivo	Functions	Reference
PANI/PVPS	NPs	808, 2 W/cm^2^	*S. aureus*, *E. coli*	No	PTT	[94]
NMPA/CS	Hydrogel	808, 0.5 W/cm^2^	MRSA	Yes	PTT	[29]
PANI/GCS	NPs	808, 0.6 W/cm^2^	MRSA *E. coli*	Yes	Targeting, Imaging, PTT	[95]
PANI/polymer	NPs	785, 0.5 W/cm^2^	*P. aeruginosa*	No	Imaging	[96]
PANI/PLNP/GCS	NPs	808, 1.5 W/cm^2^	*S. aureus*, *E. coli* MRSA	Yes	Targeting, Imaging	[97]
